# A Brief Description of How Teachers Experience An Infographic Loneliness Toolkit About Supporting Adolescents to Overcome Loneliness

**DOI:** 10.1177/13591045231209353

**Published:** 2023-11-07

**Authors:** Helena Adam, Maria Loades, Vuokko Wallace

**Affiliations:** Department of Psychology, 1555 University of Bath, Bath, UK

**Keywords:** adolescents, loneliness, teachers, schools, infographics

## Abstract

Loneliness is a common experience amongst adolescents. As adolescents spend much of their time in school, it is important that school staff can support adolescent students experiencing loneliness. The current study aimed to explore teachers’ experiences of a 1-page loneliness toolkit regarding adolescent loneliness. An online survey to collect ratings and descriptions of experiences was distributed to secondary school teachers. Findings showed that both self-rated knowledge and experience of students experiencing loneliness were positively correlated with how useful teachers found the loneliness toolkit. Three themes were developed about how useful teachers found the toolkit; clarity, brings attention to loneliness, and communication. Two themes were developed about how the user experience of the toolkit could be improved; education, and interactive student support. Future research should investigate more effective methodologies aimed at supporting adolescents experiencing loneliness to aid teachers in supporting their students.

## Introduction

Loneliness is an anguished phenomenon experienced by an individual when they have scarcity in the quality and quantity of their social relationships (Perlman & Peplau, 1981). Young people are particularly vulnerable to experiencing loneliness given that adolescence is a time when developmentally, they desire a deeper more personal connection with peers as they transition from childhood dependence on family towards independence as an adult and thus have a greater need for intimate relationships outside the family (Heinrich & Gullone, 2006). Loneliness in adolescents worldwide was found to have doubled in 2018 from 2012 (Twenge et al., 2021). Approximately 40% of UK adolescents reported experiencing loneliness at least quite frequently (Hammond, 2022). Loneliness is associated with anxiety and depression in adolescents (Loades et al., 2020).

Some ways young people in the UK cope with their loneliness include seeking to form new relationships with other individuals, denying feelings of loneliness by choosing to exclude themselves from social situations and seeking support from family, friends and professionals (Tagomori et al., 2022). A meta-analysis by Eccles and Qualter (2020) found that individual-based and group-based interventions carried out by mental health professionals were effective at reducing loneliness in young adults with no particular intervention being significantly more effective than the other.

However, providing therapeutic support to all adolescents experiencing loneliness is not likely to be possible, and as most spend considerable time in school (Verhoeven et al., 2018), school staff can be a potentially valuable source of support for adolescents experiencing loneliness. Adolescents prefer teachers offering active-based coping strategies such as support groups and emotion-based coping strategies, to support them with loneliness (Galanaki, 2004).

Little is known about how teachers can acquire resources to aid them in supporting adolescents who are lonely. Infographics, which are representations provided visually with the aim of simplifying and explaining information (Smiciklas, 2012), offer an easy way to distribute important information widely in an accessible way. In the current study, we aimed to explore the user experience of a 1-page infographic toolkit (Loades et al., 2021), developed for adults who support adolescents experiencing loneliness. The objectives were:

(a) how self-rated knowledge and experience of students experiencing loneliness is associated with how useful teachers find the loneliness toolkit.
(b) how teachers experience the toolkit
(c) how the user experience of the toolkit could be improved.

## Method

The current study utilised a mixed methods approach, with data collected using an online survey design.

### Participants

Participants were secondary school teachers from the UK who had either left their job or were currently working in schools. Participants were recruited via emailing secondary schools in the UK using contact details from the relevant school websites and advertising the study on social media platforms such as Twitter, Facebook and Instagram. A total of 58 teachers commenced the survey, and 37 completed it in full. Only the data from completers was used as those who did not complete it were assumed to have withdrawn their consent to participate.

### Measures and Materials

#### Initial Demographic Questions

Participants were asked to provide their age, ethnicity, gender identity, role within the school they worked at and how long they had been working in that role.

#### User Experience of the Loneliness Toolkit

Participants were then provided with a link which directed them to the loneliness toolkit on a separate page. The loneliness toolkit is a single-paged infographic providing information on the definition of loneliness, how it relates to the lockdown during the pandemic and ways of coping with it (https://www.bath.ac.uk/publications/loneliness-and-reconnection-guide/attachments/loneliness-reconnection-guide.pdf).

#### Quantitative Ratings

The online survey consisted of five quantitative survey items (see Appendix C). Four items measured how knowledgeable teachers felt they were in the area of students who were experiencing loneliness, how much experience teachers have had in supporting students who were experiencing loneliness during their career, how confident teachers felt in their ability to support students who were experiencing loneliness, and how useful teachers found the loneliness toolkit. Items were rated respectively as such; ‘not knowledgeable at all’ to ‘extremely knowledgeable’, ‘none at all’ to ‘a great deal’, ‘not confident at all’ to ‘extremely confident’, and ‘not useful at all’ to ‘extremely useful’. The fifth item rated how likely teachers were to use ideas from the loneliness toolkit in practice with their students. This item was rated using a Likert scale from 0 to 100. A higher score indicated a greater likelihood of teachers using ideas from the loneliness toolkit in practice with their students.

#### Qualitative Data

We developed five open-ended questions with free text boxes to capture what teachers thought of the toolkit, what teachers thought was useful about the toolkit, how teachers would apply ideas from the toolkit in practice, how the toolkit could be improved, and what else was helpful to teachers in supporting young people who were lonely in a school context.

### Procedure

This study was open to recruitment from April to July 2022. Twitter was primarily used to share study adverts, and emails were also sent to several secondary schools in the UK, asking them to share the study advert with their staff. Potential participants could click on the link provided which would direct them to the Qualtrics survey. They began the survey by reading the study information sheet, and assuming they wished to proceed, consenting to take part, followed by study measures and materials. After completing the survey, they consented again to submitting their responses, and were then able to sign up to win one of two Amazon vouchers worth £25.

### Data Analysis Plan

#### Quantitative

Descriptive statistics and graphs were used to describe the sample. As the data for self-rated knowledge/experience of students experiencing loneliness and how useful teachers find the loneliness toolkit was non-linear, Spearman’s rank-order correlation was used to test the association between these pairs of variables.

#### Qualitative

Thematic analysis (TA) was used to analyse the qualitative data. TA was chosen because it is the most suitable analysis for studies that aim to interpret meaning from the data provided (Alhojailan, 2012), allowing for a flexible yet rich account of data analysis (Braun & Clarke, 2006). Responses to questions were analysed as whole instead of individually as the findings or interpretations to a particular research question can be obtained from a range of survey questions instead of a specific survey question (Braun et al., 2020). We took a positivist approach towards the research as we intended for the analysis of the data to remain objective and be analysed systematically (Peck & Mummery, 2017). An inductive approach towards data analysis was taken to ensure all possible findings and interpretations of the data were included (Azungah, 2018). An inductive approach also helps maintain reflectivity and ensures that themes and meanings potentially established by the researcher before data analysis were avoided (Braun & Clarke, 2006).

For the research question ‘How do teachers experience the loneliness toolkit?’, analysis was combined across these three survey questions; ‘Please tell us, in your own words, what you think of this toolkit’, ‘What was useful about the toolkit?’ and ‘How do you think you will apply the ideas from the toolkit in practice?’.

For the research question ‘How could the toolkit and its user experience be improved?’, analysis was combined across these two survey questions; ‘How could we improve the toolkit and the user experience of it?’ and ‘What else is helpful to you in supporting young people who are lonely in the school context?’.

## Results

**Figure 1.**
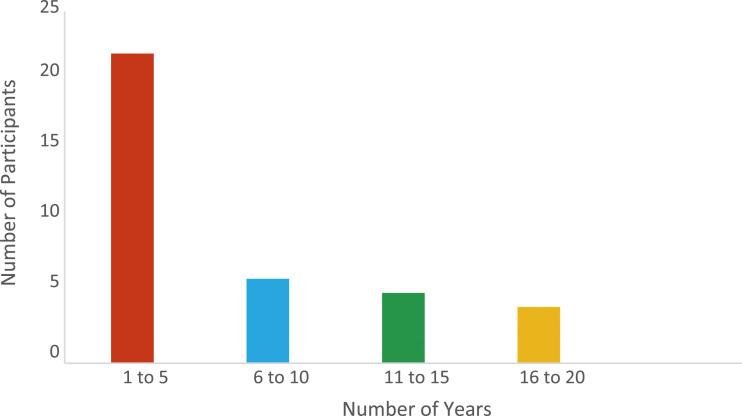
Number of years participants had been working in schools overall.

Most participants (60%) had been working in schools overall for less than 6 years (see Figure 1).

Table 1 shows the remainder of the descriptive statistics of participants based on the quantitative section of the survey.

**Table 1.** Frequency Table (number of participants endorsing each self-rated response option).

	Teachers' Self-Rating				
Number of participants	Not at all	Slightly	Moderately	Very	Extremely
Knowledge in the area of students experiencing loneliness	3	6	10	11	7
Experience in supporting students that are experiencing loneliness	2	7	12	2	14
Confidence in their ability to support students experiencing loneliness	3	2	15	12	5
Usefulness of the loneliness toolkit	0	8	10	13	6

Most participants (76%) felt at least moderately knowledgeable about students experiencing loneliness. Most (76%) felt moderately to extremely experienced in supporting students experiencing loneliness and most (86%) felt at least moderately confident in their ability to support students experiencing loneliness. Most (78%) rated the loneliness toolkit as at least moderately useful, with none rating it at not useful at all.

**Figure 2.**
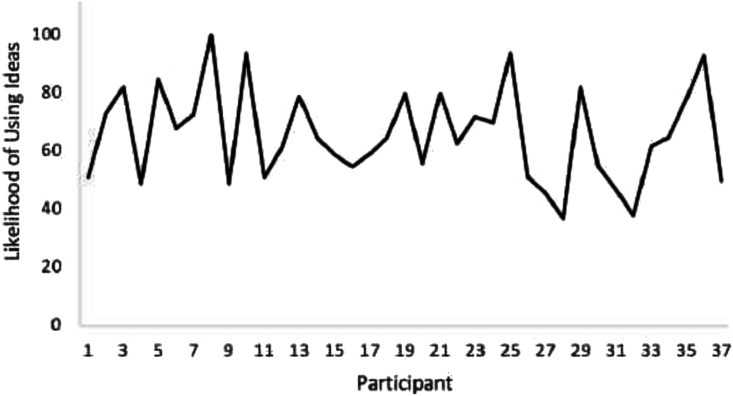
Likelihood of using ideas from the loneliness toolkit.

Figure 2 displays how likely participants are to use ideas from the loneliness toolkit in practice with their students, with 84% of participants selecting a rating of more than 50 indicating a higher likelihood to use ideas.

### Research Question: Explore How Self-Rated Knowledge and Experience of Students Experiencing Loneliness is Associated With How Useful Teachers Find the Loneliness Toolkit

There was a moderate positive correlation between the self-rated knowledge and how useful teachers found the loneliness toolkit which was statistically significant (rs (35) = .508, *p* = .001). A Spearman’s rank-order correlation was also run to determine the association between There was a weak positive correlation between experience of students experiencing loneliness and how useful teachers found the loneliness toolkit which was statistically significant (rs (35) = .326, *p* = .049).

### Research Question: How Do Teachers Experience the Loneliness Toolkit?

Three themes and seven subthemes were generated (see Table 2).

**Table 2.** Themes, Subthemes and Example Quotes From Participants Regarding Their Experience of the Loneliness Toolkit.

Themes	Subthemes	Example quotes
Clarity	Informative	Address the perception of loneliness in youth
	Practical	Easy to use, simple page, easy to understand
	Concise	The design is simple and easy to understand
Brings attention to loneliness	Visually appealing	Visually I think it is really appealing and eye catching!
	Brings awareness	Make people realize that loneliness is a constant occurrence
Communication	Conveyable to students	Can help us better help students
	Useful for staff	I think the toolkit is very helpful for my work

#### Clarity

Participants found the loneliness toolkit to be highly informative. The toolkit provided participants with a greater understanding of loneliness from the viewpoint of adolescents. One participant mentioned that the toolkit was “suitable for beginner adults who just started learning to support students that feel lonely”. They found the toolkit to be useful but more so for individuals that are in the introductory stages of learning about loneliness in adolescents. Participants also found the loneliness toolkit to be practical and concise. They found the design of the toolkit to be simple to use and easy to understand. One participant mentioned that the toolkit was “a handy guide, connecting things I sort of know already but properly researched”. They found the toolkit to be useful in reiterating known ideas in a more succinct manner to be better understood by other individuals.

#### Brings Attention to Loneliness

Participants found the loneliness toolkit to be visually appealing. The design of the toolkit was well-received by participants for its attractive aesthetics. One participant mentioned that they found the toolkit to be “really appealing” and “eye catching”. Participants also found that the loneliness toolkit brings awareness to the issue of loneliness in adolescents. One participant mentioned that the toolkit was important in “reminding us that young people can feel like this, even if they may not clearly know how to show it”. They found the loneliness toolkit useful in bringing attention to loneliness in young people, an occurrence which may not always be clearly visible or easy to identify.

#### Communication

Participants found the information in the loneliness toolkit to be easily communicated towards both students and other teachers. One participant mentioned that the loneliness toolkit “will make me more mindful to engage those students who look lost, to help them to feel ‘seen'”. They found that the toolkit provided information to not only identify adolescents experiencing loneliness, but also to support them. This would show adolescents that loneliness is not an issue which can be overlooked but one that requires being addressed and supported. Another participant mentioned that they would share the knowledge from the loneliness toolkit “with staffs that has minimum knowledge in loneliness”. They found that the toolkit had useful information they could utilize to educate other colleagues with low knowledge and awareness of loneliness. This would allow the staff to better support adolescents experiencing loneliness.

### Research Question: ‘How Could the Toolkit and its User Experience Be Improved?’

Two themes and five subthemes were generated (see Table 3).

**Table 3.** Themes, Subthemes and Example Quotes From Participants Regarding Their Suggested Improvements of the Loneliness Toolkit.

Themes	Subthemes	Example quotes
Education	Identifying more solutions	Talk about actual methods of helping, eg: talk about loneliness but in depth and why are they feeling how they felt
	Main emphasis on solutions	more focus/highlight on the possible solutions - make this the emphasis of the poster
	Life examples	Share my personal experience and let them know that all of us are lonely in some way
Interactive student support	Media	It can be turned into an animated cartoon, so that it can be shown directly to the students
	Activities	Circle time, friendship groups, specific sessions on emotional regulation and PHSE underpinning around friendships

#### Education

Participants found that identifying more solutions would enhance the usefulness of the loneliness toolkit. They suggested adding more methods of alleviating loneliness in adolescents. One participant mentioned that “There are only a couple of ideas, I would like to see more things to use”. They found the current information in the toolkit useful but wished to see more solutions regarding methods to support loneliness in adolescents. Participants also found that placing the main emphasis of the toolkit on solutions to alleviate loneliness in adolescents would be beneficial in improving the usefulness of the toolkit. They found that a toolkit should have more focus on ideas and suggestions aimed to support adolescents experiencing loneliness. One participant mentioned that they wished “to see more ideas, more opportunities to develop friendships, social skills and so on for it to be a toolkit”. They found that for a document to be classified as a loneliness toolkit, it needed to have more activities tailored towards supporting adolescents experiencing loneliness. Participants also suggested incorporating life examples in the toolkit to further increase its usefulness. One participant mentioned that they would “share my personal experience and let them know that all of us are lonely in some way.” They found that letting adolescents know about personal experiences from life would allow adolescents to relate better and understand that they are not isolated due to their issues with loneliness.

#### Interactive Student Support

Participants found that having interactive student support would further increase the usefulness of the loneliness toolkit. This can include media and activities which would directly support adolescents experiencing loneliness. One participant suggested “You can also do some fun activities.” Participants further suggested activities including showing students a cartoon based on the loneliness toolkit for them to be able to enjoy and learn in a student-friendly manner. Participants also suggested friendship groups and support sessions which would enhance supporting adolescents experiencing loneliness.

## Discussion

This exploration of teachers’ experiences as users of a loneliness toolkit for adults who support young people found that both self-rated knowledge and experience of students experiencing loneliness were positively correlated with how useful teachers found the loneliness toolkit. Teachers thought that the toolkit’s clarity, attention to loneliness, and communication contributed to its usefulness. They suggested that it could be improved further by education, such as placing more emphasis on solutions to alleviate loneliness, along with interactive student support, which included friendship groups and support sessions.

A teacher’s role besides education, is to foster a safe and nurturing environment for their students, to allow them to positively progress academically and psychologically (Garza et al., 2014). As loneliness is often difficult for teachers to identify (Morin, 2020), it would logically follow that teachers with more experience and knowledge would better understand and recognize when a student is experiencing loneliness and may also want to increase their knowledge on adolescent loneliness so they can further support their students. It is also important to consider how to engage those teachers who do not have pre-existing knowledge and experience to see the relevance of learning more about adolescent loneliness, given how common loneliness is in this age group.

It is promising that teachers perceived the loneliness toolkit as useful. One factor they mentioned was that it was clear, and the information in the toolkit was informative, practical and concise. Clarity is an important factor in the visual presentation of information. Quispel et al. (2018) found that having high clarity in the presentation of information allowed the reader to better understand information being displayed and therefore engaged the reader better in reading and processing the information. Teachers also perceived the toolkit to be useful in bringing attention towards the subject of adolescent loneliness. It may be that other school staff (e.g. administrators, pastoral care staff) and other adults who support young people outside of school (e.g. community-based workers, parents) would also perceive this toolkit to be useful, and this merits further investigation.

Teachers liked that the loneliness toolkit was easy to share with students and other members of staff. This is highly beneficial as other teachers that are not well-versed in the subject of adolescent loneliness are therefore able to learn from the toolkit and then apply those ideas in practice to support their students. This would increase the amount of support available to students experiencing loneliness. It is also important that the ideas from the toolkit are easily conveyed to students as previous research has shown that students are able to understand the idea of loneliness and are also able to suggest methods to support those experiencing it (Galanaki, 2004). These students would then be better receptive towards suggested methods that they can understand, which would not only help alleviate symptoms of loneliness in students experiencing it, but also foster a greater bond between teacher and student.

Teachers suggested that the user experience of the toolkit could be further enhanced by identifying and placing more emphasis on solutions targeted to support adolescent loneliness. A meta-analysis (Eccles & Qualter, 2020) of interventions for loneliness in young people included 39 studies of single-group designs and randomized control trials, and found that there are interventions with evidence of effectiveness in reducing loneliness. Interventions targeting emotional and social skills were most effective in single-group designs whereas interventions targeting the discovery of a new hobby were most effective in randomized group trials. However, there remains a lack of robust evidence, particularly about what works for reducing loneliness in community-based settings like schools. As the evidence base of effective interventions for adolescent loneliness develops, it will be important for toolkits like this to be updated to present the best possible advice to teachers.

Teachers also suggested that the user experience of the toolkit could be further enhanced by using examples from life. Micallef and Newton (2022) showed that using concrete examples greatly improves the learning of abstract concepts. Abstract concepts are concepts which do not possess physical forms or references (Harpaintner et al., 2018). Loneliness is an example of an abstract concept. Therefore, the use of concrete examples by teachers can further enhance adolescents’ understanding of loneliness and how to cope with it. This is because concrete examples allow definitions and information to be easily understood and retained (Weinstein et al., 2018).

Other ideas teachers had about how they could support young people experiencing loneliness included having interactive student support such as media and activities. These included circle time and friendship groups. This is consistent with prior work by Galanaki (2004) who found that students also preferred if teachers would carry out activities such as support groups as a method to support students experiencing loneliness. Previous research has found that engaging with greater social support and creating more opportunities for social contact are strongly effective in reducing loneliness (Masi et al., 2010). Therefore, this could be an important future direction for research.

### Strengths and Limitations

The online survey method of gathering qualitative data was useful as a greater number of participants could be reached and a wider range of responses allowed for a greater depth of data exploration (Braun et al., 2020). All participants opted in, meaning that they may have a particular interest in loneliness and may not be fully representative of teachers more widely. We do not know how the 21 teachers who initiated the survey but did not complete it (and were therefor excluded from the analysis) compared to those who did fully complete the survey. These participants may have had a different perception of the toolkit. Furthermore, the online survey method of collecting qualitative data meant that we could not probe for meaning, whereas in-person interviews would have enabled us to do so. As we focused on user experience, actual changes in practice by teachers as a result of the loneliness toolkit were not measured and therefore, the effectiveness of the toolkit is not clear. Given that the majority of teachers who participated in the current study were more than moderately knowledgeable and experienced in the subject of adolescent loneliness, it will be important to explore and incorporate the views of those who are less knowledgeable in future studies.

### Clinical Implications

Our findings suggest that, infographics can be an accessible, well-received method of distributing evidence based psychological information like adolescent loneliness amongst teachers. As adolescent loneliness is highly prevalent, it is important that methods such as infographics are further evaluated and then, assuming they are effective, implemented in schools to ensure teachers are provided with evidence-based resources and information in a digestible format which allows them to support their students in ways that work.

### Future Research

Future research should investigate the actual changes in practice of teachers as a result of the loneliness toolkit to understand how effective the toolkit is. Future research should also aim to explore further methods that can be used by schools and teachers to support young people experiencing loneliness as this is a highly under-researched subject (Jefferson et al., 2021). Further developing the loneliness toolkit with input from young people with lived experience of loneliness could provide valuable insight as to what might be most beneficial in supporting them with loneliness. Future research should also aim to investigate the effectiveness of these methods in generating changes in practice by teachers.

## Conclusion

The present study aimed to explore how teachers experienced a one-page infographic presenting evidence-based information about how to support young people who are lonely. We found that teachers liked the toolkit, and that it was particularly useful to those with greater knowledge and experience in adolescent loneliness. The toolkit was perceived to be accessible and useful by teachers as a means of distributing information regarding adolescent loneliness. This was due to the toolkit’s clarity, focused attention on the topic of loneliness and the ease with which information can be conveyed to others. Based on user experience feedback, we conclude that brief, visually presented information could be a useful means of informing teachers about research findings and therefore of maximising the reach and potential impact of such findings in real world practice.

## Supplemental Material

Supplemental Material - A Brief Description of How Teachers Experience An Infographic Loneliness Toolkit About Supporting Adolescents to Overcome Loneliness Supplemental Material for A Brief Description of How Teachers Experience an Infographic LonelinessToolkit About Supporting Adolescents to Overcome Loneliness by Helena Adam, Maria Loades and Vuokko Wallace
