# Supplementary material for: A Brief Description of How Teachers Experience An Infographic Loneliness Toolkit About Supporting Adolescents to Overcome Loneliness
Source: Clin Child Psychol Psychiatry. 2023 Nov 7;29(3):1058–69. doi: 10.1177/13591045231209353 (PMC11188548; doi:10.1177/13591045231209353)
Supplement: Supplemental Material - A Brief Description of How Teachers Experience An Infographic Loneliness Toolkit About Supporting Adolescents to Overcome Loneliness [file sj-pdf-1-ccp-10.1177_13591045231209353.pdf]

Appendix C

Quantitative Survey Questions

Q27 *

Using the scale below, please can you indicate **how knowledgeable you feel** you are in the area of students who are experiencing loneliness?

- ○ Extremely knowledgeable
- ○ Very knowledgeable
- ○ Moderately knowledgeable
- ○ Slightly knowledgeable
- ○ Not knowledgeable at all

Q29 *

Using the scale below, please can you indicate **how confident you fee**l in your ability to support students who are experiencing loneliness?

- ○ Extremely confident
- ○ Very confident
- ○ Moderately confident
- ○ Sightly confident
- ○ Not confident at all

Q26

Using the scale below, please indicate **how useful** you think this toolkit is?

- ○ Extremely Useful
- ○ Very Useful
- ○ Moderately Useful
- ○ Slightly Useful
- ○ Not Useful at all

Q33

Please rate how likely you are to use these ideas in practice with your students.

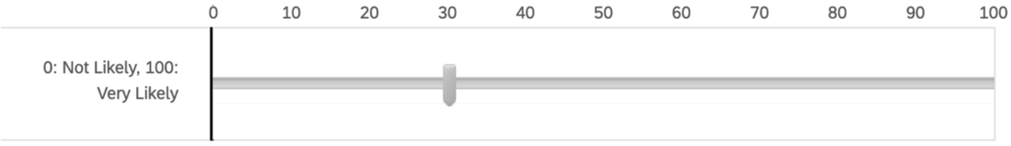

Q28 *

Using the scale below, please can you indicate **how much experience** you have had in supporting students who are experiencing loneliness during your career?

- ○ A great deal
- ○ A lot
- ○ A moderate amount
- ○ A little
- ○ None at all
